# Pathophysiological Insights into Cardiovascular Health in Metabolic Syndrome

**DOI:** 10.1155/2012/320534

**Published:** 2012-07-16

**Authors:** Yingmei Zhang, James R. Sowers, Jun Ren

**Affiliations:** ^1^Center for Cardiovascular Research and Alternative Medicine, University of Wyoming, Laramie, WY 82071, USA; ^2^Department of Cardiology, Xijing Hospital, Fourth Military Medical University, Xi'an 710032, China; ^3^Division of Endocrinology and Metabolism, University of Missouri School of Medicine, Columbia, MO 65212, USA

Obesity, diabetes mellitus, dyslipidemia, and hypertension often cluster together as the most significant risk factors for cardiovascular diseases [1–3]. Even a modest increase in body weight may lead to a rather significant increase in the prevalence of cardiovascular morbidity and mortality. Ever since its appearance in the medical vernacular three decades ago, the term “metabolic syndrome” or “cardiorenal metabolic syndrome” has caught sufficient attention due to its unfavorable impact on the overall human health in particular cardiovascular diseases. In fact, metabolic syndrome is not a particular disease but rather a cluster of symptoms encompassing large waist circumference, hypertension, hyperglycemia, dyslipidemia, and insulin resistance, all commonly associated with the increased prevalence of obesity and type 2 diabetes mellitus [3]. Patients with metabolic syndrome display nonspecific symptoms, obesity, and a sedentary lifestyle. Ample of epidemiological, clinical, and experimental evidence has defined a unique role of insulin resistance and central obesity for the occurrence of metabolic syndrome and, later on, type 2 diabetes [3, 4]. However, confirmed metabolic syndrome provides little therapeutic value or guidance other than treating each single component individually, which may or may not reduce the overall cardiovascular risks. To better address the health care issues of metabolic syndrome, intensive effort has been geared towards elucidation of the contributing cardiovascular risk factors and how they contribute to metabolic syndrome. Several contributing factors have been identified for the onset and development of metabolic syndrome. In particular, food intake and lifestyle are perhaps the most devastating factors responsible for the rapid rise in the prevalence of obesity and metabolic syndrome [5]. Excessive caloric intake and inadequate physical activity are deemed the modern lifestyle traits responsible for overweight and obesity [6]. In addition, socioeconomic factors also play an important role in the ever-increasing prevalence of metabolic syndrome including physical exercise, technological advances, and higher workload. Therefore, there is a critical need for a better understanding of the mechanisms responsible for regulation of satiety, lipid metabolism, energy balance, thermogenesis, adiposity, and weight gain, as well as central and peripheral regulation of metabolic processes. Given that recent advances in biology and medicine have introduced new technologies to study the genetics and pathophysiology of metabolic syndrome. Knowledge and understanding of these conditions lead to the development of animal models, successful therapies, and novel concepts to characterize the cardiovascular complications in metabolic syndrome, which should help to greatly improve the efficacy of clinical therapies for management of metabolic syndrome.

This special issue examines some of the critical issues in our understanding of the pathophysiology of metabolic syndrome and its cardiovascular complications. The paper by J. Palios and colleagues discusses the association between human immunodeficiency virus (HIV) and metabolic syndrome. HIV and the highly active antiretroviral therapy (HAART) may contribute to the onset of metabolic syndrome. These authors suggest a role of adipokines such as visfatin, apelin, and vaspin in the pathogenesis of the HIV/HAART-related metabolic syndrome, which should shed some lights for cardiovascular therapeutic strategy in HIV patients. The paper by N. Martinelli and coworkers addressed the rising role of low plasma concentrations of paraoxonase (PON1) in metabolic syndrome. PON1 activities are found lower in patients with metabolic syndrome, with a progressively decreasing trend by increasing the number of metabolic syndrome components. These findings suggest a role of low PON1 concentrations in predicting metabolic syndrome-associated cardiovascular diseases. The paper by I. Isordia-Salas and colleagues found a rather high prevalence of metabolic syndrome (59.7% and 68.7% based on definitions of American Heart Association/National Heart, Lung, and Blood Institute (AHA/NHLBI) and the International Diabetes Federation (IDF), resp.) in an urban Mexican sample, alerting the necessity for early detection of metabolic syndrome and its components to prevent type 2 diabetes and atherothrombotic complications in these patients. In the paper by D. Bharadwaj and coworkers, the variant gene MTHFR-rs1801133 involved in homocysteine metabolism was found to be associated with type 2 diabetes, postload glucose, high-density lipoprotein cholesterol, and total cholesterol in Indians. the paper by, S.-C. Chen and colleagues assessed the determinants of left ventricular mass index (LVMI) and left ventricular ejection fraction (LVEF) in diabetic patients at various stages of chronic kidney disease, and their findings suggest increases in LVMI and decreases in LVEF coincide with advances in chronic kidney disease stages in diabetic patients. the paper by H., Upur and colleagues reported an overtly higher prevalence of diabetes in periodontitis patients than individuals without periodontitis, suggesting the role of moderate periodontitis as an independent risk factor for type 2 diabetes mellitus.

In the second part of this special series involving basic science reports, J. R. Sowers and colleagues discussed the role of chronic alcohol consumption in cardiac insulin resistance. Chronic alcohol consumption inhibits mTOR/S6K1 activation in cardiac tissue. These authors suggested the activation of mTOR/S6K1 regulated by the mTOR-AT2R loop and microRNA that target S6K1. The paper by L. Brown and colleagues tested the therapeutic potential pf soluble epoxide hydrolase inhibition in obesity and metabolic dysfunction using a rat model of diet-induced metabolic syndrome. These authors reported that chronic oral treatment with an epoxide hydrolase inhibitor alleviates signs of metabolic syndrome including glucose, insulin, and lipid abnormalities, changes in pancreatic structure, systolic blood pressure, cardiovascular structural and functional abnormalities, and structural and functional changes in the liver. Then, L. Li and colleagues evaluated the effect of dietary supplementation of short-chain fatty acid propionate on cardiac contractile dysfunction in an Akt2 knockout-induced insulin resistance model, revealing the beneficial role of propionate or short-chain fatty acids against Akt2 deficiency-associated cardiac anomalies. The last but not least, L. Cai and colleagues review the unfavorable impact of diabetes mellitus on ischemic-preconditioning- (IPC-) and ischemic-postconditioning- (Ipost-) mediated myocardial protection. Diabetes has been shown to inhibit IPC- and Ipost-mediated myocardial protection through inhibition of the PI3K/Akt-GSK-3*β* pathway. To this end, activation of PI3K/Akt-GSK-3*β* pathway may relief the diabetic inhibition of both IPC and Ipost-mediated myocardial protection during ischemia/reperfusion injury.

As with all areas of cutting-edge human and experimental research, the advances in the pathophysiology of cardiovascular complications in metabolic syndrome described in this special raise as many questions as they answer. First, we need to better understand how individual component of metabolic syndrome work in concert to trigger cardiovascular complications. In particular, the clinical value of these individual factors in the diagnosis and treatment of cardiovascular anomies needs further scrutiny. Second, effective intervention for achieving and maintaining a healthy weight remains a major challenge for health care. Third, experimental model reminiscent of human metabolic syndrome remains a critical issue to advance metabolic syndrome research. Despite the availability of animal models for individual metabolic syndrome component, special caution is needed to translate findings from experimental setting to the clinic.

In summary, the metabolic syndrome research agenda remains a lengthy one with a large number of important questions to be answered. We hope that this special issue will help clarify the research agenda and so provide a launching pad for future progress in the field.

*Yingmei Zhang* *Yingmei Zhang* *James R. Sowers* *James R. Sowers* *Jun Ren* *Jun Ren*
